# Therapeutic Potential of Biochanin A in Herpes Simplex Keratitis

**DOI:** 10.3390/ph16091240

**Published:** 2023-09-01

**Authors:** Nan Zhou, Deyuan Zheng, Qiao You, Taige Chen, Jiaxuan Jiang, Wenhao Shen, Di Zhang, Junpeng Liu, Deyan Chen, Kai Hu

**Affiliations:** 1 Department of Ophthalmology, The Affiliated Drum Tower Hospital, Medical School of Nanjing University, Nanjing 210008, China; mg21350088@smail.nju.edu.cn (N.Z.);; 2 State Key Laboratory of Pharmaceutical Biotechnology, Chemistry and Biomedicine Innovation Center, Medical School of Nanjing University, Nanjing 210093, China; 3 Center for Public Health Research, Medical School of Nanjing University, Nanjing 210093, China; 4 Nanjing Drum Tower Hospital Clinical College, Nanjing University of Chinese Medicine, Nanjing 210023, China

**Keywords:** HSK, biochanin A, HSV-1, anti-viral, anti-inflammatory, anti-oxidative stress, anti-apoptotic

## Abstract

Herpes simplex keratitis (HSK) is a blinding eye disease that is initiated by the herpes simplex virus type 1 (HSV-1). Resistance to acyclovir (ACV) and the side effects of corticosteroid drugs have become concerning issues, so it is crucial to develop new antivirals for treating HSK. In this study, we report that biochanin A (BCA), a naturally occurring flavonoid compound, provides multifaceted protective effects with anti-viral, anti-inflammatory, anti-oxidative stress and anti-apoptotic activities to alleviate HSK. The results show that BCA significantly inhibited HSV-1 replication in vitro and further proved that BCA principally influenced the early stage of virus infection. We reveal that BCA downregulated the expression of pro-inflammatory factors triggered by HSV-1, including TNF-α, RANTES, IL-1β and IL-6. Furthermore, BCA treatment alleviated oxidative stress and apoptotic arising from HSV-1 infection. Lastly, we induced HSK in male C57BL/6 mice and treated them with either BCA or phosphate buffer solution (PBS) eye drops. We observed the ocular surface lesions; determined the virus load in the tear fluid, corneas as well as trigeminal ganglions (TGs); and detected the levels of inflammation and apoptosis in the corneas simultaneously. These results show that BCA inhibits HSV-1 and alleviates the corneal lesion degree. Our study illustrates that BCA is a promising therapeutic approach for application in treating HSK.

## 1. Introduction

Herpes simplex virus type 1 (HSV-1), an enveloped dsDNA virus, can replicate in human epithelial cells and establish latent infection in neurons [1]. The virus is highly prevalent and endemic throughout the world [2,3], with the majority of the world’s population reportedly living with this virus [4]. Infection due to HSV-1 is usually asymptomatic, but it can also cause severe diseases, such as keratitis and encephalitis [5]. Herpes simplex keratitis (HSK), which primarily affects eyelids, corneas, or conjunctiva, is an important infectious cause of blindness worldwide [6]. The current standards of care include topical corticosteroids and antivirals. Topical corticosteroid therapy is usually used to reduce inflammation to control serious infection, but it can lead to numerous sequelae, and antivirals are only beneficial if replicating virus is present [7,8,9]. More importantly, the resistance of HSV-1 to acyclovir is a problem of growing clinical importance [10]. Thus, there is an urgent need to develop new anti-herpetic drugs and strategies.

Typically, when HSV-1 infects a cornea, it starts virus replication in the corneal epithelial cells, activates the innate immune system followed by the adaptive one, and increases the production of inflammatory substances, including cytokines and chemokines [11]. Although it is conducive to controlling viral infections, immune responses are major contributors to corneal lesions [12]. An immune-mediated inflammatory reaction leads to HSK, which results in corneal thinning, focal stromal opacity, corneal neovascularization (CNV), corneal scarring, and possibly blindness [13]. Consequently, it is an ideal HSK treatment approach to inhibit viral replication as well as reduce ocular inflammation, which is the detrimental effect of immune responses.

**Figure 1:**
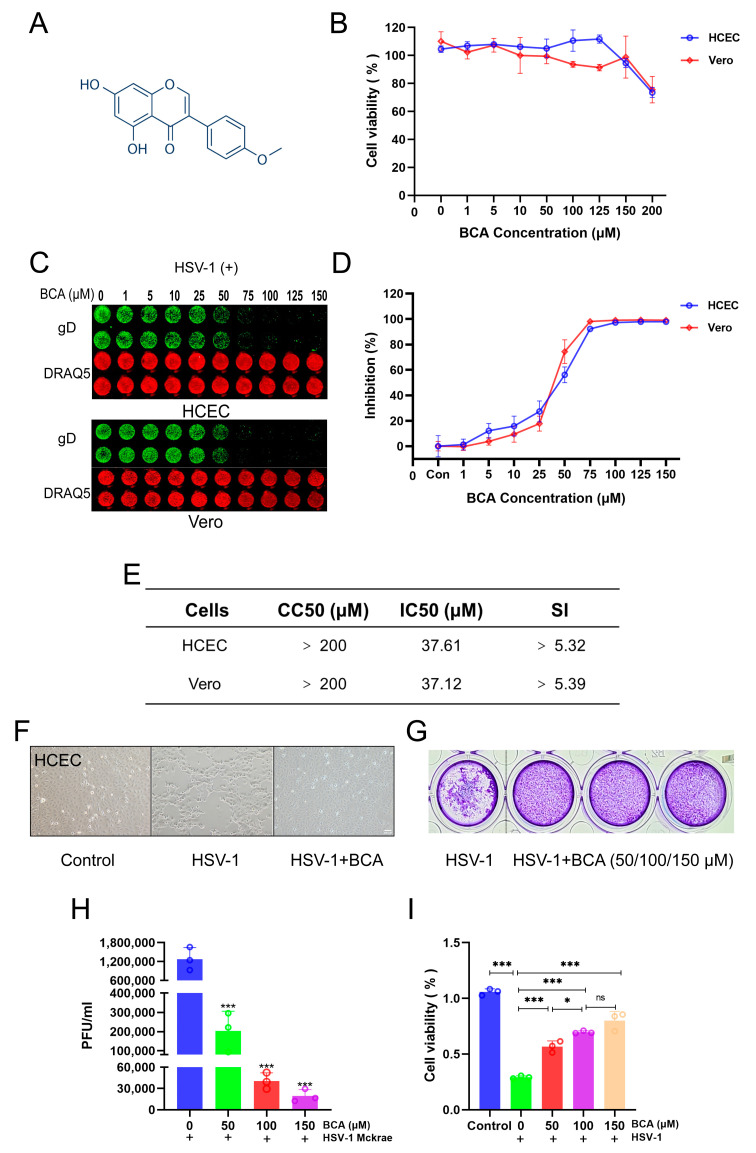
BCA suppressed HSV-1 replication in HCECs and Vero cells. (**A**) The basic structure of biochanin A. (**B**) Cell viability of HCECs and Vero cells exposed to BCA at different concentrations for 24 h. (**C**,**D**) HCECs and Vero cells were added with dilution HSV-1 and treated with various doses of BCA for 24 h. In-cell Western analysis was used to identify the viral protein glycoprotein D (gD, green), which was normalized by DRAQ5 (red). The viral inhibition curves were determined via the fluorescence expression of gD protein. (**E**) CC50, the half-maximal cytotoxicity concentration; IC50, the half-maximal inhibitory concentration; SI, selectivity index (SI = CC50/IC50). Information is available in the table. (**F**) HCECs were infected with HSV-1 and treated with vehicle or BCA (150 μM). The cell monolayers were photographed at 24 h post-infection (hpi) (10×). (**G**) Inhibition of BCA on plaque formation. (**H**) Vero cells were planted in 12-well plates and infected with HSV-1 (MOI = 1) in the existence of BCA for 24 h. The half-maximum tissue culture-infective dose (TCID50) was used to calculate viral titers in the media. (**I**) Cell viability of HCECs exposed to HSV-1 and treated with BCA for 24 h. The data were presented as mean ± SD of at least three independent experiments (* *p* < 0.05 and *** *p* < 0.001).

Natural compounds are a prominent source of novel antiviral drugs [14]. Biochanin A (4′-methoxy-5, 7-dihydroxy isoflavone, BCA) (Figure 1A) is a dietary isoflavone extracted from the leaves and stems of Trifolium pratense L and several other Chinese medicine herbs [15]. The pharmacological and biological activities of BCA are well-documented as anticancer [16,17], antioxidant [18], and its marked anti-inflammatory effects [19]. In terms of antiviral effects, BCA has only been reported to have anti-H5N1 activity [20]. Notably, in silico findings revealed that BCA might also be an effective treatment against SARS-CoV-2 [21]. As a result, less is understood about BCA’s antiviral capabilities in the treatment of infectious disorders. In this paper, we present the first proof that BCA inhibits HSV-1 replication in vitro and in vivo and has a protective effect on HSK.

## 2. Results

### 2.1. BCA Inhibited HSV-1 Replication In Vitro

We initially investigated BCA’s cytotoxicity by treating HCECs and Vero cells with varying doses of BCA for 24 h before assessing cell viability using the CCK8 assay. According to Figure 1B, BCA exhibited no obvious cytotoxicity in cells at concentrations below 200 μM. However, treatment with 200 μM BCA elicited about 30% cell death and thus we chose doses of less than or equal to 150 μM to use in subsequent experiments. Then, the inhibitory impact of BCA on HSV-1 was assessed. HCECs and Vero cells were infected with HSV-1 while being treated with escalating doses of BCA. After 24 hpi, the gD protein expression was assessed with an In-Cell Western assay, and the IC50 values were calculated concurrently. As illustrated in Figure 1C,D, BCA significantly inhibited HSV-1 infection at 75–150 μM concentrations. For BCA, the 50% cytotoxic concentration (CC50) was more than 200 μM, and the IC50 was determined to be about 37 μM (Figure 1E). Accordingly, the biologically active concentration was significantly lower than the cytotoxicity one. As shown in Figure 1F, in the presence of BCA or a solvent control, HCECs were infected with HSV-1 (MOI = 1). At 24 hpi, the cytopathic effect was clearly observed in vehicle-treated cells, but not in BCA-treated cells. Subsequently, 50, 100 and 150 μM were chosen as concentrations of BCA treatment in the following in vitro experiments. The antiviral activity of BCA was analyzed by a standard plaque assay as the gold standard phenotypic method. Compared to the control group, those treated with BCA produced significantly fewer plaques (Figure 1G). BCA also significantly reduced viral particles in the culture supernatants (Figure 1H). Furthermore, BCA at concentrations ranging from 50 to 150 μM protected HCECs from death induced by HSV-1 (Figure 1I). Taken together, our data show that BCA inhibited HSV-1 infection in vitro.

### 2.2. BCA Suppressed the Expression of HSV-1-Immediate–Early (IE), -Early (E) and -Late (L) Genes and Blocked HSV-1 at an Early Stage

**Figure 2:**
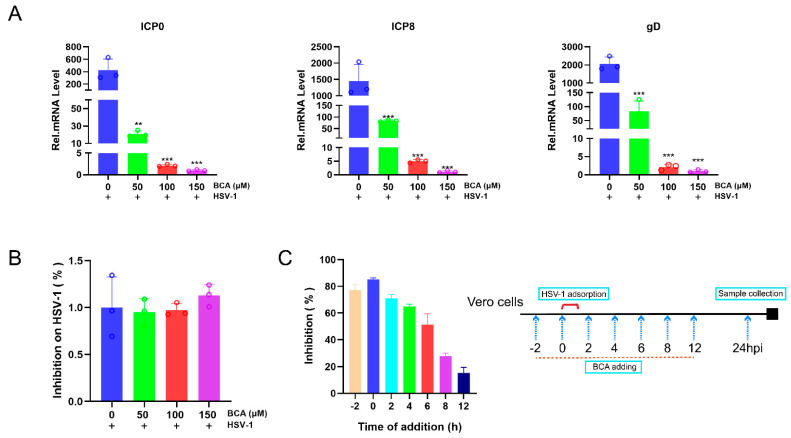
Effects of BCA on the expression of HSV-1 genes and replicative circle. (**A**) Vero cells were infected with HSV-1 and then treated with 50, 100 or 150 μM BCA. ICP0, ICP8 and gD mRNA transcription levels at 24 hpi were determined via qRT-PCR. (**B**) BCA has no virucidal effect against HSV-1. (**C**) Vero cells in 24-well plates were infected with HSV-1, and then 150 μM BCA was added at specified time points (−2, 0, 2, 4, 6, 8 and 12 hpi). We used the samples at 24 hpi (without BCA) as a control to reflect the viral replication. Culture mixtures were collected at 24 hpi. The virus load was analyzed by qRT-PCR. The time-of-drug-addition assay revealed that the inhibition of BCA turned out to be more effective in the early stage of HSV-1 infection. The data were presented as mean ± SD of at least three independent experiments (** *p* < 0.01 and *** *p* < 0.001).

To gain further insight into BCA’s effects on HSV-1 infection, we investigated whether BCA prevented HSV-1 replication by modulating the expression of replication-associated viral genes. ICP0, ICP8 and gD corresponded to the IE, E and L genes, respectively, and were necessary for HSV-1 replication [22,23,24]. According to Figure 2A, the relative mRNA levels of IE genes ICP0, ICP8 and gD were significantly decreased in the presence of BCA for 24 h in a dose-dependent manner. Therefore, BCA suppressed HSV-1 replication by inhibiting the viral IE, E and L genes. Following that, virucidal experiments were conducted to rule out the idea that the antiviral action against HSV-1 was due to the direct inactivation of the released virus. HSV-1 viral suspensions were mixed with different doses of BCA for 2 h at 37 °C before being diluted to a non-inhibitory drug concentration and introduced into Vero cells. After 24 h, we used qRT-PCR to detect viral gene expression. There was no difference in HSV-1 gene expression between the control and BCA groups (Figure 2B). This implies that the pre-treatment with BCA has no virucidal effect on HSV-1 virions. We used the time-of-addition assay to identify which phase of the HSV-1 replication cycle BCA inhibited. Vero cells were exposed to 150 μM BCA at varied time intervals of −2, 0, 2, 4, 6, 8 and 12 hpi. After 24 hpi, viral gene expression was detected by qRT-PCR. As shown in Figure 2C, HSV-1 replication was significantly inhibited when BCA was added at −2, 0 and 2 hpi. As seen in Figure 2C, when BCA was introduced at −2, 0 and 2 hpi, HSV-1 replication was noticeably suppressed, while the antiviral efficacy steadily diminished with the time lag of intervention. This finding indicates that BCA might have a role in the beginning of HSV-1 replication.

### 2.3. The Inhibition of HSV-1 Infection by BCA Was Independent on Interferons (IFNs) and BCA Reduced the Overproduction of Inflammatory Cytokines in Corneal Epithelial Cells

**Figure 3:**
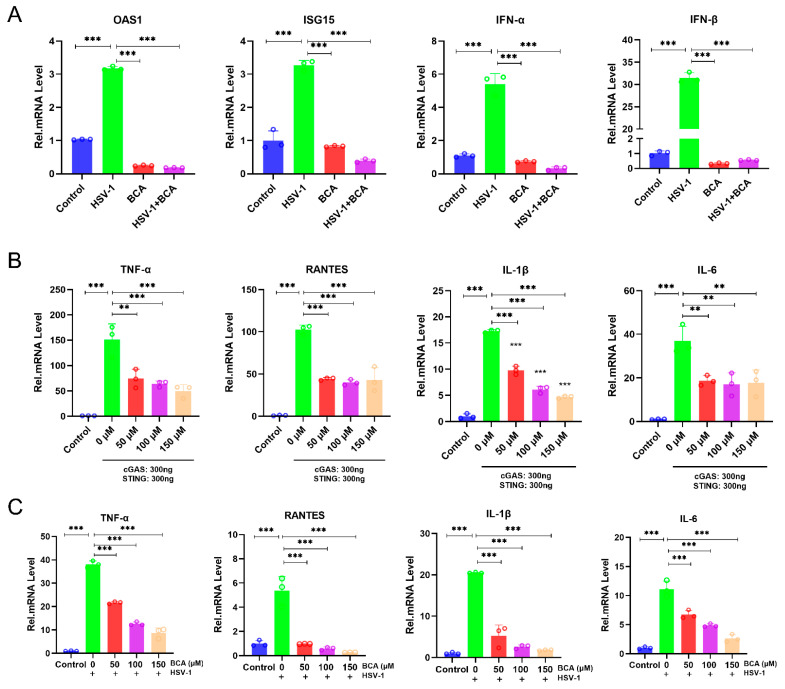
The effects of BCA on the expression of IFNs and IFN-regulated genes and the anti-inflammatory property of BCA in the context of HSV-1 infection. (**A**) Levels of OAS1, ISG15, IFN-α and IFN-β were analyzed in HCECs by qRT-PCR. (**B**) Following BCA treatment for 24 h, 293 T cells were transfected with FLAG-cGAS plus FLAG-STING or the corresponding mutant plasmids, and gene expression was assessed by qRT-PCR. As a negative control, an empty vector was transfected. (**C**) BCA reduced the transcription of TNF-α, RANTES, IL-1β and IL-6 genes induced by HSV-1 infection for 12 h in HCECs in a dose-dependent manner. The data are presented as mean ± SD of at least three independent experiments (** *p* < 0.01 and *** *p* < 0.001).

Next, we determined whether BCA could increase the expression of type I IFN and interferon-induced genes. OAS1, ISG15, IFN-α and IFN-β mRNA were not activated by BCA in HCECs treated with BCA (Figure 3A). Therefore, BCA had a non-interferon-mediated action against the virus in corneal epithelial cells, which might contribute to the lower release of inflammatory mediators. We then sought to validate the anti-inflammatory activity of BCA in the context of HSV-1 infection. Beforehand, we confirmed the anti-inflammatory property of BCA. We transfected cultured 293 T cells with cGAS and STING expression plasmids and then treated them with BCA. Then, we measured the transcription of TNF-α, RANTES, IL-1β and IL-6. Our data show that BCA inhibited the transcription of genes linked to the inflammatory response triggered by the overexpression of cGAS and STING (Figure 3B). The inhibitory effects of BCA on HSV-1-induced inflammation were further investigated via an established HSV-1-infected HCEC model. The results suggest that BCA inhibited the HSV-1 infection-triggered activation of TNF-α, RANTES, IL-1β and IL-6 proinflammatory genes in a dose-dependent manner in HCECs (Figure 3C).

### 2.4. BCA Alleviated Oxidative Stress and Apoptosis in Corneal Epithelial Cells Infected by HSV-1

**Figure 4:**
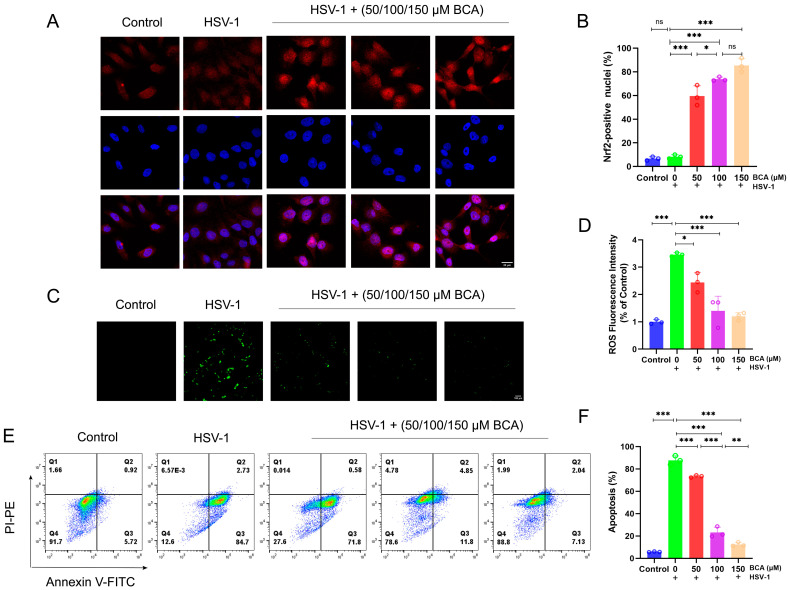
BCA augmented Nrf2 nuclear translocation, reduced the production of ROS and inhibited the apoptosis in infected HCECs. (**A**) Immunofluorescence staining of Nrf2 and DAPI colocalization in HCECs. (**B**) Quantification of Nrf2-positive nuclei in each group. (**C**) Fluorescence microscopy was used to monitor the generation of ROS in each treatment group. (**D**) A fluorescence microplate reader for detecting the fluorescence intensity of ROS. (**E**) The apoptosis rates of HCECs in different treatment groups were examined by flow cytometry using Annexin-V FITC/PI staining. (**F**) Quantification of apoptosis rates of HCECs in different treatment groups (*n* = 3). The data are from at least three independent experiments (* *p* < 0.05, ** *p* < 0.01 and *** *p* < 0.001).

According to previous research, HSV-1 infection produces oxidative stress in tissues and cells [25]. Remarkably, Nrf2 activation is recognized as a targeted method for regulating oxidation [26]. As shown in Figure 4A,B, we investigated the localization and expression (red fluorescence intensity) of Nrf2, and immunofluorescence staining showed that the BCA-treated group significantly motivated the activation of Nrf2 and its translocation to the nucleus. Moreover, an excess of reactive oxygen species (ROS) resulted in oxidative stress, which is one of the key elements in the pathogenesis of corneal disorders [27]. According to previous studies, HSV-1-infected cells could induce the production of ROS, which is beneficial to viral replication and leads to oxidative damage. Considering the notable antioxidant properties of BCA, we wondered whether BCA could prevent HSV-1-induced ROS overproduction. HCECs were infected with HSV-1 (MOI = 1) after BCA pretreatment. The intracellular ROS level at 24 hpi was detected by immunofluorescence microscopy (Figure 4C) and quantified by measuring the level of fluorescence on a microplate reader (Figure 4D), following DCFH-DA staining. HSV-1 infection significantly increased fluorescence in HCECs compared to the control cells, but BCA treatment weakened fluorescence intensity and removed ROS.

It has been reported that the activation of apoptotic pathways may be necessary for efficient viral replication in corneal epithelial cells [28]. We also examined the apoptosis rate of HCECs in different treatment groups by flow cytometry with Annexin-V FITC/PI staining. In the results of flow cytometry, early apoptotic and late apoptotic cells were located in the lower right and upper right quadrants, respectively. The sum of cells in both quadrants represents all apoptotic cells. The data indicate that HSV-1 infection increased the percentage of apoptotic cells in cells, while BCA decreased the apoptotic rate (Figure 4E,F).

### 2.5. Safety Assessment of Topical BCA on the Cornea in Mice

**Figure 5:**
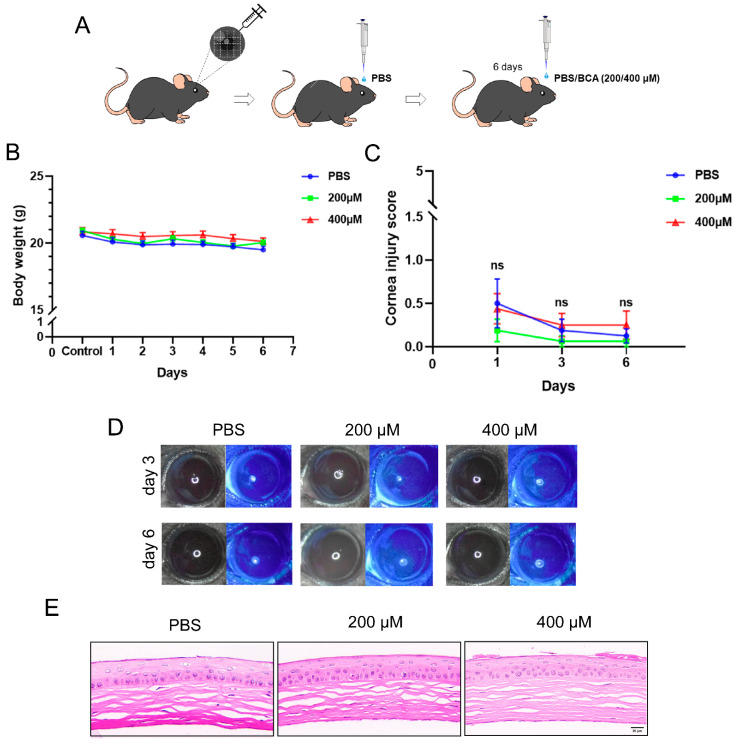
The toxicity of BCA on the cornea in mice. (**A**) Schematic of the experiment. (**B**) Mice’s relative body weights were recorded for six days in a row after being scratched (*n* = 7). (**C**) Corneal injury scores were recorded according to the grading system for corneal opacity. PBS or topical BCA was administered to the model eyes three times each day. On days 1, 3 and 6, these corneas were checked, and the corneal injury scores were noted. (**D**) Typical images of fluorescein sodium staining taken on days 3 and 6. (**E**) H&E staining of mouse corneal sections on day 6.

The toxicity of BCA for the corneal epithelium was assessed prior to conducting mice models of efficacy tests. First, right eyes were scarified with a 30-gauge needle and then 5 μL PBS was dropped on the cornea (Figure 5A). This intervention was to simulate the infection process and reflect the impact of medication on the ocular surface more accurately. Next, mice were randomly divided into three groups and received topical application of PBS or doses of BCA (200 μM and 400 μM) higher than therapeutic one (150 μM) three times per day for 6 days. We recorded daily body weight and observed the ocular surface lesions by fluorescein sodium staining on day 1, 3 and 6 for each mouse. The data suggest that there were no significant differences between the three groups in body weight during administration (Figure 5B). Compared with PBS-treated mice, corneal transparency and epithelial integrity were not affected (Figure 5C,D). We further examined damage in corneal structure by HE staining (Figure 5E), and there were no changes in the BCA-treated groups. These results suggest that it is safe to use BCA eye drops within a certain range of concentration in mice eyes.

### 2.6. BCA Treatment Ameliorates HSK Severity in Mice

**Figure 6:**
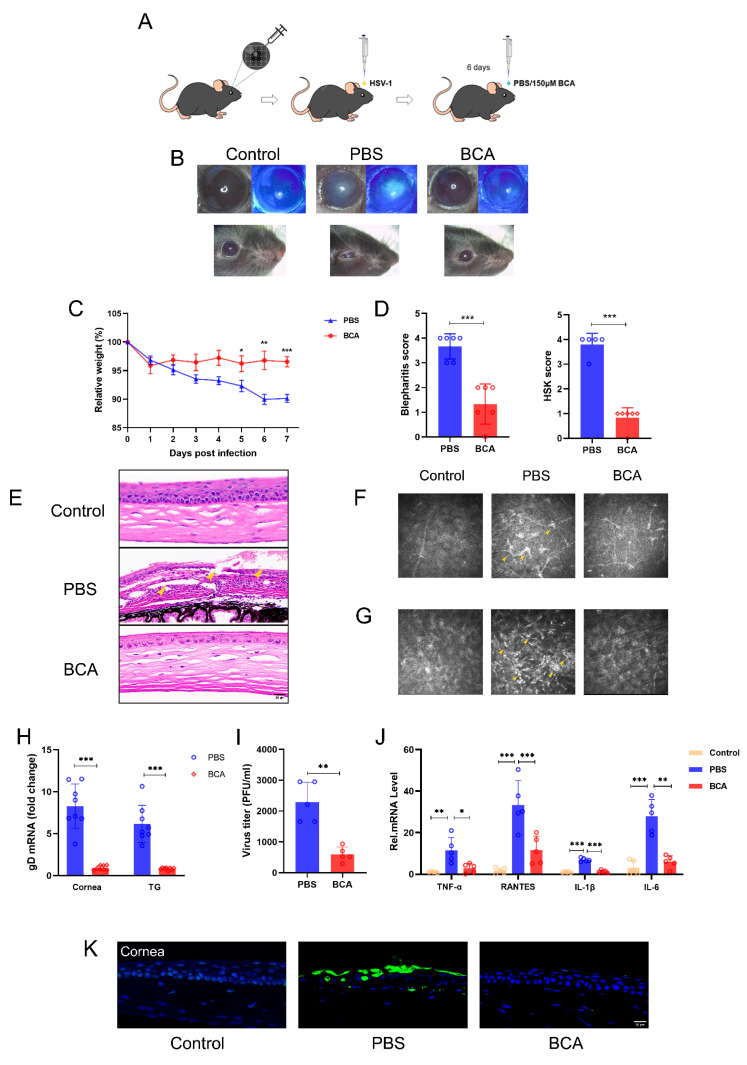
Topical application of BCA reduced HSK in vivo. (**A**) Drug delivery procedure after 5 × 5 ocular injuries with a needle. (**B**) Representative photographs of mice’s right eyes were captured at 6 dpi. (**C**) The body weights of mice were monitored for 7 days after HSV-1 infection (*n* =  6). (**D**) Scores of blepharitis and corneal lesions in mice at 6 dpi (*n* = 6). (**E**) H&E staining was performed to obtain corneal pathological changes at 6 dpi. (**F**,**G**) The mature LCs (with typical branching dendrites) and keratocyte activation (with higher reflectivity) of the PBS group were significantly higher than those of the BCA group (arrowhead). (**H**) qRT-PCR was utilized to examine the mRNA transcripts of HSV-1 gD in mice corneas and TGs at 6 dpi, with GAPDH serving as an internal control (*n* = 8). (**I**) The secreted virus titers were measured from the right eyes of mice at 6 dpi (*n* = 5). (**J**) The expression levels of TNF-α, RANTES, IL-1β and IL-6 in cornea tissues were measured by qPCR (*n* = 5). (**K**) TUNEL staining showing the apoptotic cells in the corneal epithelium of mice in each group. The data are presented as mean ± SD of at least three independent experiments (* *p* < 0.05, ** *p* < 0.01 and *** *p* < 0.001).

To investigate the therapeutic effects of BCA in vivo, mice were separated into two groups and received a topical application of BCA (150 μM) or PBS three times for 6 days after HSV-1 infection (Figure 6A). We chose the 6 dpi to observe, photograph and collect the samples. Compared to the BCA-treated group, the PBS-treated group had obvious corneal opacification, serious blepharitis and even difficulty in eye opening (Figure 6B). By comparison, BCA treatment greatly reduced HSV-1-related eye symptoms. Following HSV-1 infection, body weights were measured daily. We noticed a statistically significant reduction in body weight in the PBS-treated mice compared to the BCA-treated ones after 5 dpi (Figure 6C). Mice treated with BCA displayed significant improvement in clinical symptoms as measured by using the blepharitis score and the HSK lesion degree score (Figure 6D). The HE staining illustrated that the corneas in the PBS group were structurally affected, with an apparent loss of corneal epithelium cells and a notable influx of inflammatory cells (Figure 6E). In vivo confocal microscopy (IVCM) was applied to further observe the pathological changes in HSK. Langerhans cell (LC) maturation and keratocyte activation were the characteristic appearance of HSK by IVCM, and the former was also an excellent indicator of inflammatory activity [29,30]. LC maturation (Figure 6F) and keratocyte activation (Figure 6G) were observed evidently in the PBS group while failing to be seen in the BCA one. Moreover, we measured the virus levels in the eyes of two groups. We collected tears from the treated mice and calculated TCID50 using the method of Reed–Muench. We observed that BCA treatments obviously reduced the viral titers at 6 dpi. We evaluated the HSV-1 gene expression in tissues by qRT-PCR. At 6 dpi, gD expression was dramatically reduced in both the corneas and the trigeminal ganglions (TGs) of the BCA-treated mice (Figure 6H). Additionally, we discovered that BCA treatments clearly decreased the virus titers at 6 dpi (Figure 6I). We further detected the expression of pro-inflammatory cytokines TNF-α, RANTES, IL-1β and IL-6 by qRT-PCR. The results show that BCA eye drops inhibited the transcription of pro-inflammatory cytokines in the corneal tissues (Figure 6J). In addition, TUNEL staining was used to examine apoptosis in tissues. The results show that BCA inhibited the apoptosis of the corneal epithelium, which played a protective role (Figure 6K). These data demonstrate that BCA efficiently ameliorated HSK.

## 3. Discussion

**Figure 7:**
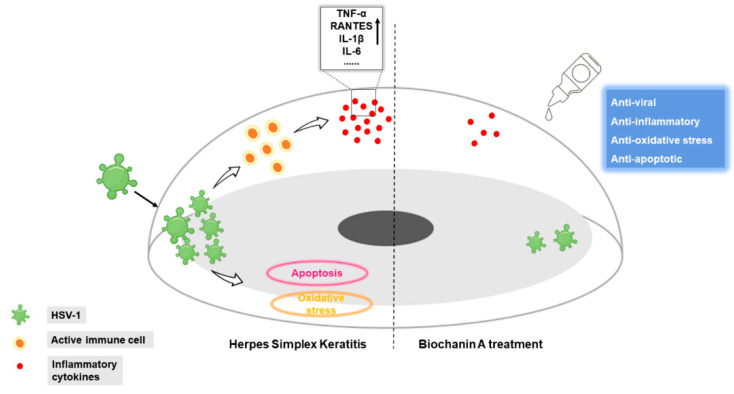
Biochanin A alleviated herpes simplex keratitis by inhibiting HSV-1 replication, downregulating inflammatory cytokines, relieving oxidative stress and decreasing the apoptosis of corneal epithelial cells.

HSV-1 corneal infection causes rapid HSV-1 replication and epithelial damage, and then the host cell contacts with HSV-1 creates an inflammatory cascade that is responsible not only for virus clearance but also for progressive corneal opacification due to inflammatory cell infiltrate, angiogenesis and corneal nerve loss. In this research, we proved that the natural flavonoid BCA has antiviral activity and therapeutic effects both in vitro and in vivo, and therefore showed enormous potential as a new treatment for HSK (Figure 7).

HSV-1 infection remains a serious worldwide health concern, particularly given the rising incidence of drug resistance in immunocompromised patients, emphasizing the importance of developing new effective treatment options [31]. To date, there has been little focus on the clinical development of biologically active natural ingredients for anti-HSV drugs. Thus, the search for new drugs derived from natural products with reduced resistance, fewer side effects and diverse mechanisms of action is critical in breaking down the barriers to novel antiherpetic drug development [32]. Biochanin A’s anti-inflammatory activity has been extensively investigated, but the study of BCA antiviral activity remains limited. In this study, we first revealed that BCA inhibited HSV-1 infection through blocking virus replication at an early stage, implicating that BCA could be a potential anti-herpetic drug candidate. In a future study, the combination inhibitory effect of BCA and acyclovir deserves further investigation to provide a better choice of treatment. Additionally, the transcript level of HSV-1 gD was decreased in the TGs after BCA treatment, which means that BCA may also play a protective role in recurrent HSK.

HSV-1 can rapidly stimulate the innate immune response in the cornea, gradually causing corneal opacification and visual loss [33]. Primarily neutrophils, macrophages and NK cells are among the innate response cells that rapidly infiltrate after the initial HSV-1 exposure [34]. Various pro-inflammatory cytokines, including interferons, TNF-α and IL-6, are secreted by innate immune cells [35,36]. Although these immune responses are intended to be protective, the cytokines generated by immune cells reduce visual acuity by causing fibrosis and inflammation [37]. It has been documented that HSV-1 can induce cGAS-STING signaling, which is an important cytosolic DNA sensor mechanism in innate immunity and an immune inflammatory route, and the activated STING initiates a sequence of downstream pathway activation and events that transcriptionally up-regulate inflammatory cytokines and other factors, such as IL-6, TNF-α and RANTES [38,39,40,41]. The hyperproduction of pro-inflammatory cytokines can drive pathological inflammation and exacerbate HSK [42]. Thus, blocking them can alleviate corneal immunopathological damage. Therefore, we simulated the process of the HSV-1-triggered activation of cGAS-STING pathway and the subsequent expression of proinflammatory cytokines by transfection. Our in vitro experiments proved that the transcription of representative cytokines (TNF-α, IL-1β, RANTES and IL-6) in 293 T cells transfected with cGAS and STING expression plasmids were downregulated by BCA. Afterwards, we demonstrated the anti-inflammatory effect of BCA in the context of HSV-1 infection on corneal epithelial cells. However, specific mechanisms that mediate the inflammation regulation of BCA in HSK still require further investigation.

Oxidative stress is often induced by viruses to overwhelm the infected cells and create an environment that favors its replication [43], and HSV-1 is no exception [44]. There is evidence that oxidative stress participates in HSK and its inhibition can protect cells against oxidative damage, thereby alleviating corneal injury induced by HSV-1 infection [45]. In addition, interactions between HSV-1 and host cells result in the triggering of the apoptotic cell death program [46]. Compelling evidence indicates that HSV-1 infection leads to increased apoptosis in both animal models and patients with ocular HSV-1 infection [47,48]. Thus, we explored and confirmed that BCA played a role in regulating oxidative stress and reducing corneal epithelium cell apoptosis in HSK.

The treatment mechanisms of BCA on HSK are still in their infancy and can be progressed in multiple aspects. Firstly, prior studies have reported that the upregulation of Nrf2 activity restricted HSV-1 viral infection [49,50], and isoflavones have the ability to activate Nrf2 [51]. Several studies have confirmed that BCA has the ability to activate Nrf2 [52,53], and our study determined it too. Therefore, we further hypothesized that BCA could inhibit HSV-1 replication via activating Nrf2 signaling pathway. Second, the modulatory effects of BCA on the inflammatory response need further studies. The bulk of cells in corneal lesions are known to be derived predominantly from neutrophils, which cause inflammatory processes as well as tissue damage, and the same is true for HSK [54]. Importantly, BCA has been reported to promote the activation of pro-resolving programs, including neutrophil apoptosis [55]. Whether this action plays a role in HSK deserves further investigation. Moreover, the roles of BCA in other immune cells and inflammatory response in HSK models equally deserve further inquiry and exploration. Finally, corneal neovascularization (CNV), one of the most serious corneal complications of HSK, is a problem that cannot be ignored [56]. Multiple studies have shown that BCA has the property of reducing neovascularization by directly targeting various facets of angiogenesis [57,58]. In a follow-up study, the effects of BCA on HSK-induced CNV will be further explored. Altogether, we need more evidence to reinforce the important therapeutic potential of BCA to pave the way for a deeper application of BCA in clinical practice. In this paper, we just explored the anti-HSV-1 activity of BCA, but there must be more than that. Due to its antiviral capabilities and powerful anti-inflammatory properties, the natural flavonoid biochanin A is a prospective option for the treatment of infectious disorders associated with inflammation.

## 4. Materials and Methods

### 4.1. Cell Culture, Viruses and Reagents

Vero cells were kindly gifted by Professor Zhiwei Wu from Nanjing University. Human SV40 immortalized corneal epithelial cells (CRL-11135, HCE-2; ATCC, Manassas, VA, USA) were cultured in DMEM/F12 medium (Gibco, Billings, MT, USA) supplemented with 10% FBS (Gibco, USA) in a humidified 37 °C 5% CO2 incubator. The HSV-1 strain McKrae was used in this study and prepared as described previously [59]. It was propagated and titrated using a plaque-forming unit (PFU) assay as previously described [60,61]. Biochanin A was purchased from MedChemExpress (Shanghai, China). Antibody to gD was brought from Santa Cruz (Santa Cruz, CA, USA).

### 4.2. Cytotoxicity Assay

HCECs or Vero cells were cultured in 96-well plates for 12 h and then treated with various concentrations of BCA. After incubation for 24 h, the supernatant was removed and a diluted CCK8 solution (Vazyme, Nanjing, China) was added to each of the wells and incubated for another 1.5 h. The optical density (OD) values were measured at 450 nm by a multimode microplate reader (Tecan Spark, Männedorf, Switzerland). Cell viability was expressed as the percent of untreated cells.

### 4.3. In-Cell Western Assay

This assay was performed by the Odyssey Infrared Imaging System (LI-COR, Lincoln, NE, USA), according to the manufacturer’s instructions. HCECs or Vero cells grown in 96-well plates were fixed and permeabilized with Triton X-100 (Beyotime, Shanghai, China), and incubated with PBS-containing 5% BSA after infection and treatment. The cells were then stained overnight at 4 °C with gD antibody (1:400). After being washed three times with PBST, the cells were stained with IRDye IgG (1:1000) for 1 h, following 1 h DRAQ5 staining and being scanned with an Odyssey Infrared Imager. The relative amount of gD protein expression was obtained by normalizing to endogenous DRAQ5 in all experiments. DRAQ5 is a far-red DNA stain for fluorescent cellular imaging applications in live cells.

### 4.4. Antiviral Activity

HCECs or Vero cells were cultured in 96-well plates and then infected with HSV-1 (multiple of infection (MOI) = 1). Simultaneously, the cells were exposed to BCA at the indicated concentrations. After 24 h, the supernatant was discarded, and the virus load was measured by the In-Cell Western assay.

### 4.5. Quantitative Real-Time PCR (qRT-PCR)

Total RNA was extracted from cells and corneal or TG tissues using TRIzol reagent (Takara, Kusatsu, Japan). The concentration and purity of the total RNA were examined by a Nanodrop 2000 (Thermo Fisher Scientific, Waltham, MA, USA). A total of 1 μg of the total RNA was reverse-transcribed using the HiScript III RT SuperMix (Vazyme, Nanjing, China) following the manufacturer’s instructions. The expression levels of the target genes were measured by qRT-PCR on QuantStudio 5 (Thermo Fisher Scientific, USA). Gene expression was normalized to that of GAPDH and determined by the 2^−ΔΔCt^ method. Primer sequences are listed in Supplementary Table S1.

### 4.6. Plaque Reduction Assay

Vero cells were pretreated with BCA for 12 h after being seeded on the plates and adhering to the wall, and then PBS was used to remove the residue. All wells were added with the virus inoculum, and the supernatant liquid was removed 1 h later. The monolayers were overlaid with a 1:1 mixture of low-melting-point agarose and 2 × MEM and placed at room temperature for 20 min until solidified. Then, the plates were incubated for 48 h to collect the samples. A total of 500 μL crystal violet was added to each hole during the sample collection and then placed in the refrigerator at 4 °C for 4 h; the gel was rinsed off and then dried.

### 4.7. Time-of-Drug-Addition Assay

Vero cells were seeded in 12-well plates (5 × 10^5^ cells/well). The cells were exposed to HSV-1 (MOI = 1) for 1 h. Next, the viral supernatant was removed, and the cells were washed three times with PBS. At −2, 0, 2, 4, 6, 8 and 12 h post-infection (hpi), 150 μM BCA was added to the infected cells. After 24 hpi, the samples of each time point were collected for viral yield measurement using qRT-PCR.

### 4.8. Virucidal Assay

Vero cells were seeded in 24-well plates to 80% confluence. Different concentrations of drugs and virus dilutions (50 PFU/well) were mixed and incubated at 37 °C for 2 h in a humidity incubator. Virus control groups were mixed with the serum-free media. After that, the mixture was diluted to non-inhibitory concentrations of the compounds and added to the plates; each concentration had three duplicate wells, and incubated at 37 °C for another 2 h. After 24 hpi, cells were collected for viral yield measurement by qRT-PCR.

### 4.9. Transfection

The 293 T cells were seeded on six-well plates. On the following day, cells were transfected with PRK-FLAG-cGAS and PRK-FLAG-STING using Lipofectamine 3000 (Thermo Fisher Scientific, USA), according to the manufacturer’s protocol.

### 4.10. Immunofluorescence

HCECs were fixed with 4% paraformaldehyde for 30 min and then permeabilized with Triton X-100 (0.5% in PBS) for 15 min at room temperature and blocked with donkey serum for 1 h. Following that, the cells were exposed to primary Nrf2 antibody (Santa Cruz) for a whole night at 4 °C. After rinsing with PBST, the cells were incubated with Alexa Fluor 568 (Servicebio, Wuhan, China) secondary antibody for 1 h at room temperature. DAPI staining was applied for an additional 5 min to make it easier to see the nuclei. Fluorescence microscopy was used to capture and evaluate images.

### 4.11. Measurement of ROS

The ROS activities of cells were measured by a fluorescent probe (DCFH-DA) (Beyotime, Shanghai, China). The HSV-1-infected HCECs were treated with BCA at certain concentrations. After 24 h, cells were subsequently incubated with DCFH-DA for 20 min and washed three times with PBS before the analysis by the multimode microplate reader (Tecan Spark, Austria) or the Leica Thunder system (Leica, Wetzlar, Germany).

### 4.12. Analysis of Apoptosis

HCECs were cultivated in six-well plates for 24 h at a density of 5 × 10^5^ cells per well. The cells were pretreated with BCA for 12 h and then infected with HSV-1 for an additional 2 h. Following the manufacturer’s instructions, the Annexin V/PI staining kit (Beyotime) was used to assess the amount of apoptosis that occurred in the treated cells. BD Accuri C6 (BD, San Jose, CA, USA) was used to detect the total number and percentage of apoptotic cells, and FlowJo V10 (FlowJo, LLC, Ashland, OR, USA) was utilized to analyze the data.

### 4.13. Mice

Male C57BL/6 mice weighing 18–20 g were acquired from Nanjing Medical University’s Animal Center in Nanjing, China. The Nanjing First Hospital’s Animal Ethics Committee provided its approval to all experimental procedures in accordance with the ARVO Statement for the Use of Animals in Ophthalmic and Vision Research (permission ID: 23017617). The animals were kept in a shared environment with constant access to food, water and air conditioning.

### 4.14. Corneal Toxicity of BCA in a Mouse Model

All mice were administered an intraperitoneal injection of 1% sodium pentobarbital (80 mg/kg) to make them unconscious. The right corneas were scraped with a 30-gauge needle and inoculated with 5 µL PBS as a control. The injured corneas were topically treated three times daily with PBS or BCA (200 μM and 400 μM) and evaluated for corneal opacity and epithelial abnormalities on days 3 and 6. Corneal opacity was assessed following the Supplementary Table S2.

### 4.15. Herpes Simplex Keratitis Mouse Model

The method of anesthesia was the same as that described above. The right corneas of mice were scarified by 5 × 5 strokes with a 30-gauge needle and were inoculated with 5 µL HSV-1 strain McKrae (1 × 10^6^ PFU/mL). The infection was carried out unilaterally. The first treatment was started at about 5 h after infection. A total of 150μM BCA (5 μL) or vehicle (PBS, 5 μL) were topically applied three times per day for 6 days to test the therapeutic effects of BCA in vivo. These eyes were inspected, eye swabs were obtained from their tears to determine virus titers and tissues at 6 days post-infection (dpi) were extracted for subsequent investigations. The severity of the disease was measured by the blepharitis score and the herpes simplex keratitis lesion extent score. Details of scoring criteria are listed in Supplementary Table S3.

### 4.16. TUNEL Assay

The eyes were separated and embedded in optimum cutting temperature (OCT) glue, sliced into sagittal sections and then kept at −80 °C. Frozen slices of mouse eyeballs were evaluated for apoptosis of corneal epithelium using an in situ cell death detection kit and fluorescein, according to the manufacturer’s recommendations. 

### 4.17. Statistical Analysis

GraphPad Prism 8.0 (GraphPad software Inc., Boston, MA, USA) was used for statistical analysis. Comparisons between two groups were identified via *t*-tests. One-way ANOVA was used to compare data among three or more groups. All results were repeated for at least three times. The data are presented as mean ± SD and were considered as statistically different when *p* < 0.05 (* *p* < 0.05, ** *p* < 0.01 and *** *p* < 0.001).

## 5. Conclusions

In conclusion, our research shows the therapeutic efficacy of BCA in HSK both in vivo and in vitro, particularly in terms of antiviral and protective activities. BCA shows considerable promise as a potential therapy for HSK and inflammatory illnesses of the ocular surface.

## Data Availability

The data presented in this study are available upon request from the corresponding author.

## Supplementary Materials

The following supporting information can be downloaded at: https://www.mdpi.com/article/10.3390/ph16091240/s1, Table S1: Primer sequences used in qPCR; Table S2: Grading system for corneal opacity; Table S3: Herpes simplex keratitis lesion extent score and Blepharitis score.
