# Supplementary material for: Therapeutic Potential of Biochanin A in Herpes Simplex Keratitis
Source: Pharmaceuticals (Basel). 2023 Sep 1;16(9):1240. doi: 10.3390/ph16091240 (PMC10536220; doi:10.3390/ph16091240)
Supplement: Supplementary file 1 [file pharmaceuticals-16-01240-s001.zip › pharmaceuticals-2548584-supplementary.pdf]

Table S1. Primer sequences used in qPCR

Gene	Forward primers	Reverse primers
HSV-1 ICP0	ATGTCTGGGTGTTTTTCCCTGC	TCTCGAACAGTTCCGTGTCC
HSV-1 ICP8	CGACAGTAACGCCAGAAG	GGAGACAAAGCCCAAGAC
HSV-1 gD	AGCAGGGGTTAGGGAGTTG	CCATCTTGAGAGAGGCATC
GAPDH (human)	AATGGGCAGCCGTTAGGAAA	GCGCCCAATACGACCAAATC
OAS1 (human)	GAGCTCCTGACGGTCTATGC	CAGTCCTCTTCTGCCTGTGG
ISG15 (human)	GTGGACAAATGCGACGAACC	ATTTCCGGCCCTTGATCCTG
IFN-β (human)	AGTAGGCGACACTGTTCGTG	AGCCTCCCATTCAATTGCCA
IFN-α (human)	ACACCAGGTCACGCTTTCAT	AGCATGGTCATAGTTATAGCAGGG
IL-6 (human)	GTCCAGTTGCCTTCTCCCTGG	CCCATGCTACATTTGCCGAAG
TNF-α (human)	CACAGTGAAGTGCTGGCAAC	AGGAAGGCCTAAGGTCCACT
IL-1β (human)	AGCCATGGCAGAAGTACCTG	TGTCCATGGCCACAACAACT
RANTES (human)	GGATCAAGACAGCACGTGGA	TCGGGTGACAAAGACGACTG
GAPDH (mouse)	TGATGACATCAAGAAGGTGGTGAAG	TCCTTGGAGGCCATGTGGGCCAT
RANTES (mouse)	CTGCTGCTTTGCCTACCTCT	CTTGAACCCACTTCTTCTCTGG

Table S2. Grading system for corneal opacity

Grade	Description
0	Completely transparent cornea
1	Minimal corneal opacity, but iris clearly visible
2	Moderate corneal opacity, iris vessels still visible
3	Moderate corneal opacity, pupil margin but iris vessels not visible
4	Complete corneal opacity, pupil not visible

Table S3.

Herpes simplex keratitis lesion extent score

Grade	Description
0	no epithelial lesions or punctate lesions, no edema or cloudiness of the stroma
1	stellate epithelial lesions or mild edema and cloudiness of the stroma
2	dendritic or atlas-like epithelial lesions covering less than 25% of the cornea, stromal edema or cloudy lesions less than one-half of the corneal diameter
3	dendritic or atlas-like epithelial lesions covering 25%–50% of the corneal area, stromal edema or cloudy lesions larger than one-half of the corneal diameter
4	dendritic or atlas-like epithelial lesions occupying >50% of the corneal area, severe stromal edema and clouding, with no visible iris

Blepharitis score

Grade	Description
0	swelling of eyelids;
1	mild swelling of the eyelids;
2	moderate swelling of the eyelids plus moderate crusting;
3	half of eyelids shut plus severe crusting;
4	eyelids completely shut
